# Effects of a Foot Pump on the Incidence of Deep Vein Thrombosis After Total Knee Arthroplasty in Patients Given Edoxaban: A Randomized Controlled Study

**DOI:** 10.1097/MD.0000000000002247

**Published:** 2016-01-08

**Authors:** Tatsuya Sakai, Masahiro Izumi, Kenji Kumagai, Kenichi Kidera, Takayuki Yamaguchi, Tomohiko Asahara, Hideko Kozuru, Yuka Jiuchi, Masaaki Mawatari, Makoto Osaki, Satoru Motokawa, Kiyoshi Migita

**Affiliations:** From the Department of Orthopedic Surgery, NHO Nagasaki Medical Center, Kubara, Omura/ Department of Molecular Immunology, Nagasaki University Graduate School of Biomedical Sciences, Sakamoto, Nagasaki.

## Abstract

We conducted a randomized clinical trial to compare the effectiveness of the A-V Impulse System foot pump for reducing the incidence of deep-vein thrombosis (DVT) after total knee arthroplasty (TKA) in patients under edoxaban thromboprophylaxis.

Patients undergoing primary TKA at our institution between September 2013 and March 2015 were enrolled after obtaining informed consent. The patients were randomized to use the foot pump (n = 58) and not to use the foot pump (n = 62). Both groups were given prophylactic edoxaban. Primary outcomes were any DVT as detected by bilateral ultrasonography up to postoperative day 10 (POD10) and pulmonary embolism (PE) up to POD28. The safety outcomes were bleeding and death of any cause up to POD28. Plasma D-dimer levels were measured before TKA and on POD10 after TKA. Immunoglobulin G (IgG)-class anti-PF4/heparin antibodies were measured using an IgG-specific enzyme-linked immunosorbent assay.

The incidences of any DVT up to POD28 were 31.0% and 17.7% in patients with or without the foot pump, respectively. The incidences of major bleeding up to POD28 were 5.1% and 4.8% in patients with or without the foot pump, respectively. Foot pump use did not significantly reduce the incidence of DVTs in patients undergoing TKA under edoxaban thromboprophylaxis. Although seroconversion of anti-PF4/heparin antibodies was confirmed in one-fourth of patients, the seroconversion rates did not differ between patients with (20.7%) or without (25.8%) foot pump use.

This study shows that the A-V Impulse system foot pump did not affect the incidence of DVT under edoxaban thromboprophylaxis in patients undergoing TKA. Seroconversion of anti-PF4/heparin antibodies was detected in a significant number of patients who underwent TKA under antithrombotic prophylaxis using edoxaban.

## INTRODUCTION

Total knee arthroplasty (TKA) is considered as a successful procedure that improves quality of life for patients with end-stage knee joint arthritis.^1^ However, the risk of venous thromboembolism (VTE), which can increase patient morbidity and mortality, is well recognized after TKA.^2^ There are evidence-based guideline to prevent VTE,^3,4^ but the ideal prophylactic regimen has not been identified. The selection of a prophylactic regimen depends on a balance between efficacy and safety.^5^ Such regimens include low-molecular-weight heparin (LMWH), Xa inhibitors, and mechanical methods such as pneumatic foot compression.^6^ Several randomized trials have shown that the foot pump is an effective device for prophylaxis against thromboembolism in orthopedic patients.

Combined prophylactic modalities have been shown to improve the efficacy of single modalities in a variety of specialties.^7–9^ Edoxaban is an oral, selective, direct factor Xa (FXa) inhibitor that was approved as a thromboemboprophylactic agent in Japanese patients undergoing total joint arthroplasty.^10,11^ We therefore aimed to compare the frequency of thromboembolism after total knee replacement in patients who were randomized to be managed postoperatively with edoxaban thromboprophylaxis (both groups) with or without use of the foot pump. We also sought to evaluate these roconversion rates of anti-PF4/heparin antibodies in these patients.

## METHODS

### Patient Enrollment

Consecutive patients (aged ≥20 years) undergoing knee replacement surgery for primary joint disease, including osteoarthritis (OA) and rheumatoid arthritis (RA) were enrolled. Exclusion criteria were the presence of predefined risk factors for bleeding, coagulation disorders, heart failure (New York Heart Association class III or IV), significant renal dysfunction (creatinine clearance <30 mL/min), and abnormalities in biochemical measurements (aspartate aminotransferase or alanine aminotransferase ≥5 times the upper limit of normal or total bilirubin ≥2 times the upper limit of normal). Patients were also excluded if they were scheduled to undergo bilateral joint replacement or reoperation, were unable to walk, or had uncontrolled cardiovascular disease.

### Study Subjects

**FIGURE 1:**
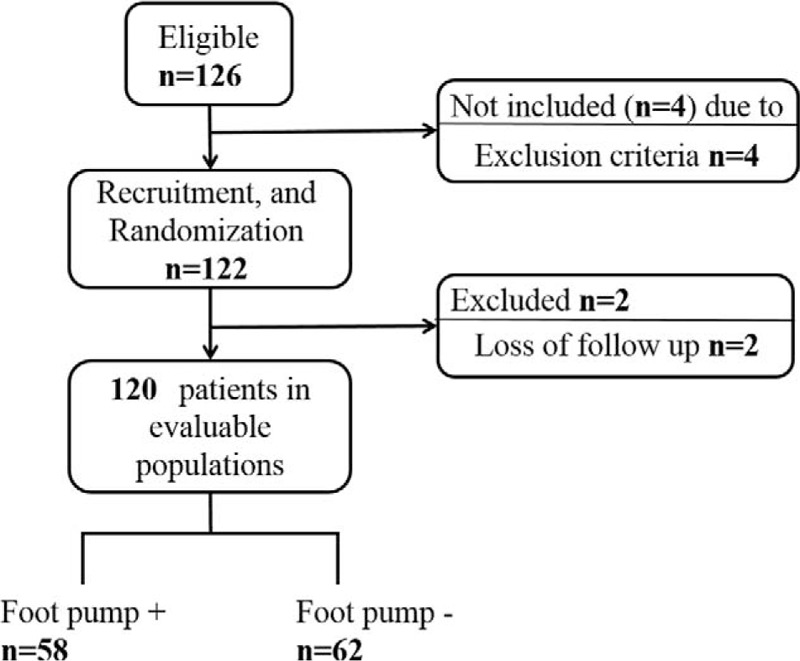
Flow Diagramof patient recruitment.

Between September 2013 and March 2015, a total of 126 patients were considered for inclusion in the study. As shown in the flow diagram (Figure 1), among these patients, 4 were excluded due to the exclusion criteria: 1 because of painful joints in the feet, which contraindicated use of the foot pump, and 3 because of contraindications for edoxaban. After enrollment, 2 patients were excluded after being lost to follow-up (Figure 1). The ethics committees at the Nagasaki Medical Center, where the study was conducted, approved the study protocol (No. 25004), and all patients signed informed consent forms.

### Randomization

Randomization was performed on the day before the operation using sealed envelopes containing a slip indicating the allocation, which had been derived from a computer-generated sequence. Patients were given edoxaban alone or edoxaban plususe of the foot pump. Edoxaban was started 12 h after the operation. Patients were given low-dose edoxaban (15 mg once daily for patients <60 kg) or high-dose edoxaban (30 mg once daily for patients weighing ≥60 kg). Foot pump slippers were fitted for both feet in the recovery room, and the machine was activated. The nurses were advised to activate the foot pump whenever the patient was not bearing weight. The nurses routinely monitored the use of mechanical compression by checking it every 3 h until POD4. An alarm was also set to sound when the foot pump turned off or pressure did not appear. The pneumatic compression cycle was set at 20/min with a pressure of 130 mm Hg applied for 1 s. All patients wore bilateral knee-high antithromboembolic stockings. Physical therapy, range of motion exercises, and walking with partial weight bearing were usually initiated on POD4. The foot pump was continued until the start of physical therapy (POD4).

### Outcome Measures

The primary effectiveness outcomes are overall venous thromboembolism (VTE) including asymptomatic DVTs up to POD10, symptomatic DVTs, and fatal/nonfatal pulmonary embolisms (PEs) up to POD28. All patients were checked for DVTs by ultrasonography (US) on POD10 or earlier if thrombosis was clinically suspected. DVT was diagnosed according to the presence of a venous thrombus detected by compression US (CUS), under the standardized method.^12,13^ DVTs were classified as proximal vein (popliteal vein and vein proximal to it) or a distal vein (any vein distal to the popliteal vein). PE was diagnosed according to the presence of intraluminal filling defect detected by computed tomography of the chest. The outcomes for safety were the bleeding and death from all causes up to POD28. Major bleeding was defined as wound hematoma or hemorrhage occurring at a critical site and bleeding required for >2 units of red blood cell concentrates. Minor bleeding was defined as bleeding that did not fulfill the criteria for major.

### Blood Sampling

Serum samples were obtained before the operation and at POD10 and were stored at −30°C. A enzyme-linked immunosorbent assay (ELISA) kit (GTI Diagnostics, Waukesha, WI) was used to measure the IgG-class antiheparin-platelet factor 4(PF4) antibody (HITantibody) according to the manufacturer's instructions. ELISA reactivity (optical density, OD) was expressed relative to a standard control. The cutoff value was set at 0.40 optical density (OD) units. We defined seroconversion as a positive test result on POD10 corresponding to a negative result before surgery if the patient's blood sample on POD 10 was positive and had a 2-fold or more increase in OD, as defined in a previous study.^14^

### Statistical Analysis

**FIGURE 2:**
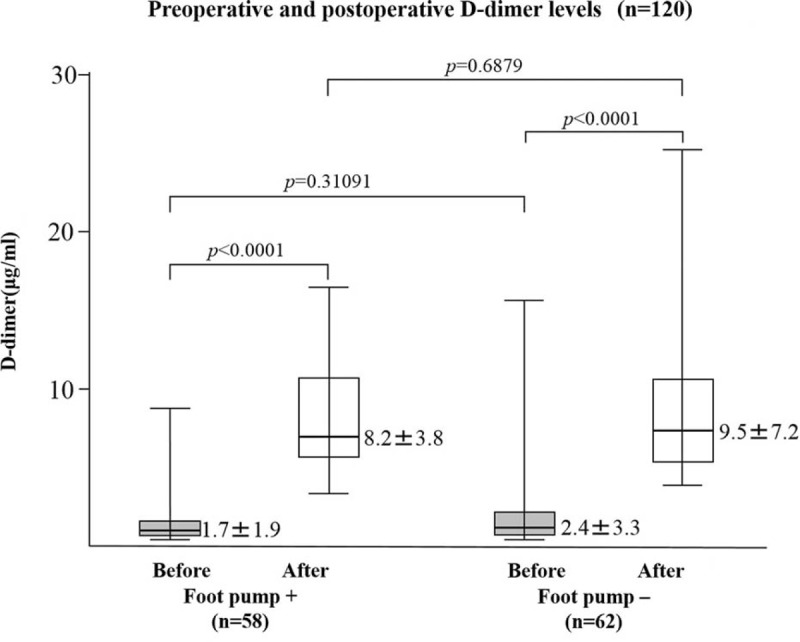
Preoperative (before) and postoperative day 10 (after) plasma D-dimer levels in patients with or without use of foot pump. *P* values were calculated by the Mann–Whitney *U* test.

Discrete variables were compared using χ^2^ tests and continuous variables using Mann−Whitney tests. Plasma D-dimer levels are expressed as means (±SD) or medians (interquartile ranges) in Figure 2. Boxplots display the lower hinge defined as the 25th percentile, the middle hinge as the 50th percentile, and the upper hinge as the 75th percentile. All reported *P* values were 2-tailed. All data processing and analyses were performed using the Statistical Analysis System (SAS) and SPSS version 18 software (SPSS, Chicago, IL).

## RESULTS

### Patient Demographic Data

The characteristics of the study population (total patients117; RA 23, OA 94) undergoing TKA are presented in Table 1. The risk of VTE in each patient was individually assessed based on the presence of the risk factors listed in Table 1, including, age, sex, and comorbidities. Of the 120 patients who were ultimately randomized, 58 used the foot pump and edoxaban, and 62 used edoxaban alone. The 2 groups were comparable in terms of their baseline characteristics and the operations they underwent (Table 2). The demographic data showed some differences between patients with or without the foot pump, but the differences did not reach statistical significance. All patients underwent pharmacological thromboprophylaxis with edoxaban. The mean doses of edoxaban were 20.4 ± 7.3 mg for 11.6 ± 1.7 days in patients with the foot pump and 21.3 ± 7.5 mg for 11.3 ± 2.0 days in those without the foot pump. Three patients received aspirin because of their ischemic heart disease (Table 2). Aspirin administration was stopped 7 days before operation and started again on POD3 in all 3 patients. There were no statistically significant differences in the doses of edoxaban use between the 2 groups.

**TABLE 1:**
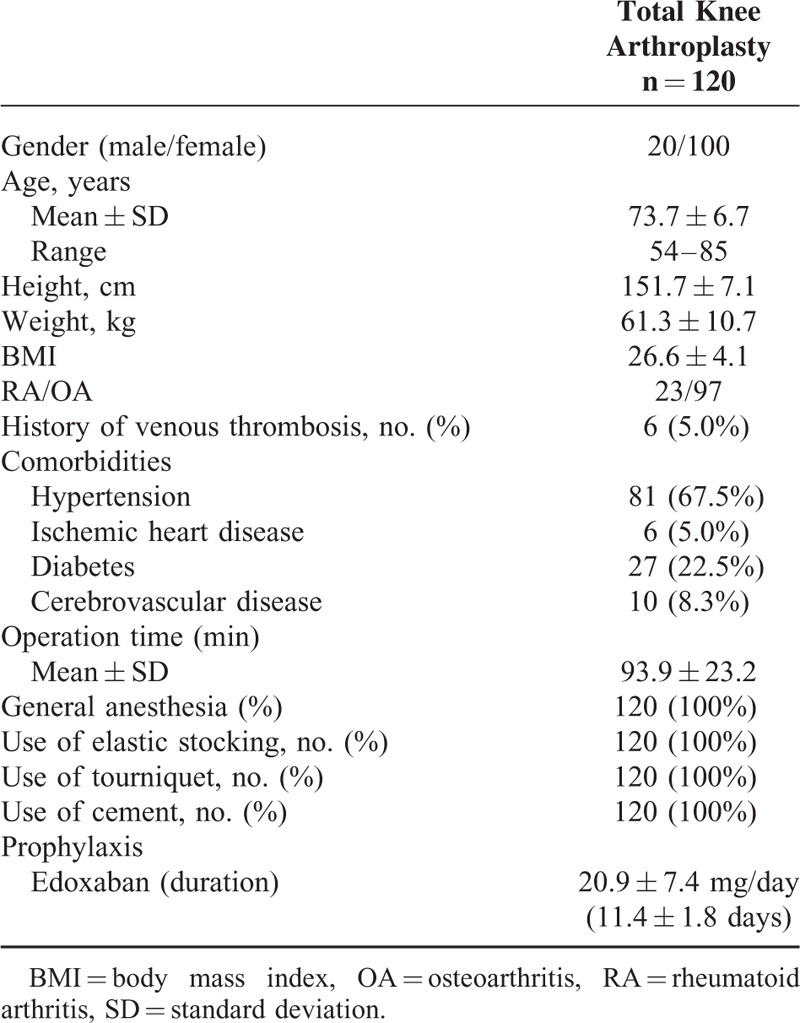
Baseline Characteristics of the Patients Receiving Total Knee Arthroplasty

**TABLE 2:**
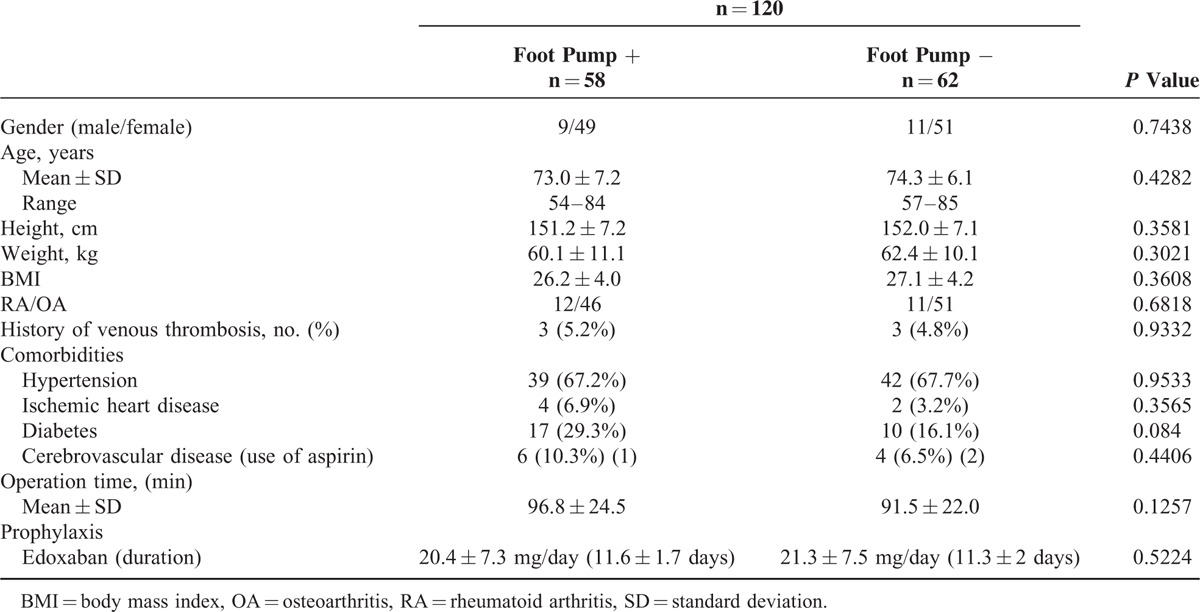
Baseline Characteristics of the Patients With or Without Foot Pump

### Incidence of Postoperative VTE

The rate of all DVT appearances up to POD10 was 31.0% (18 patients) (Table 3). Symptomatic DVTs occurred in 3 patients (5.2%) with the foot pump, and 17.7% (11 patients), with symptomatic DVTs occurring and in 1 patient (1.6%) without the foot pump. The locations of the thrombi are shown in Table 2. Most thrombi were located in the distal veins in both groups. Comparisons were also performed to identify predictors of DVTs. The demographic data showed some differences in doses of edoxaban between patients with or without DVT (Table 4). The 2 groups were otherwise similar. When we compared the incidence of postoperative total DVTs between the patients with and without use of the foot pump (Table 5), there was no significant difference in the incidence of DVTs between the 2 groups(*P* = 0.089; odds ratio [OR] 2.09, 95% confidence interval [CI] 0.89−4.87). Hence, use of the foot pump did not prevent the occurrence of DVTs.

**TABLE 3:**
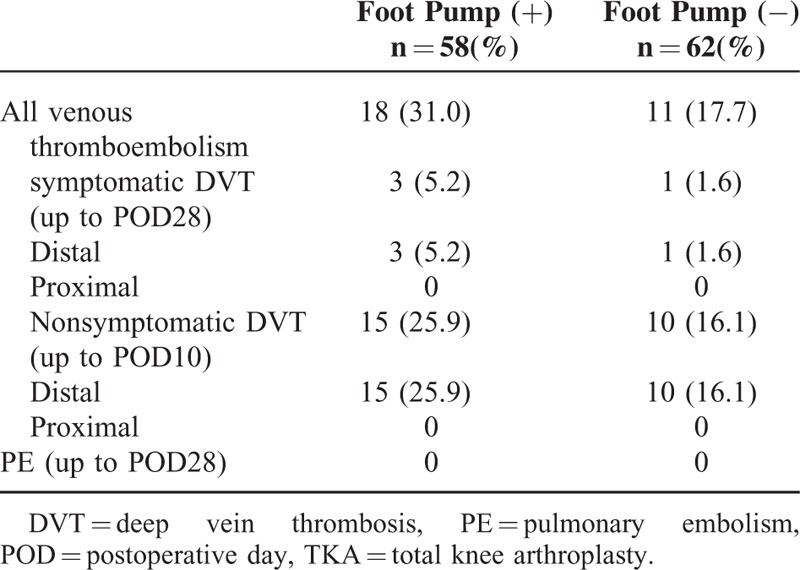
Incidences of Primary Effectiveness Outcomes in Patients Receiving TKA

**TABLE 4:**
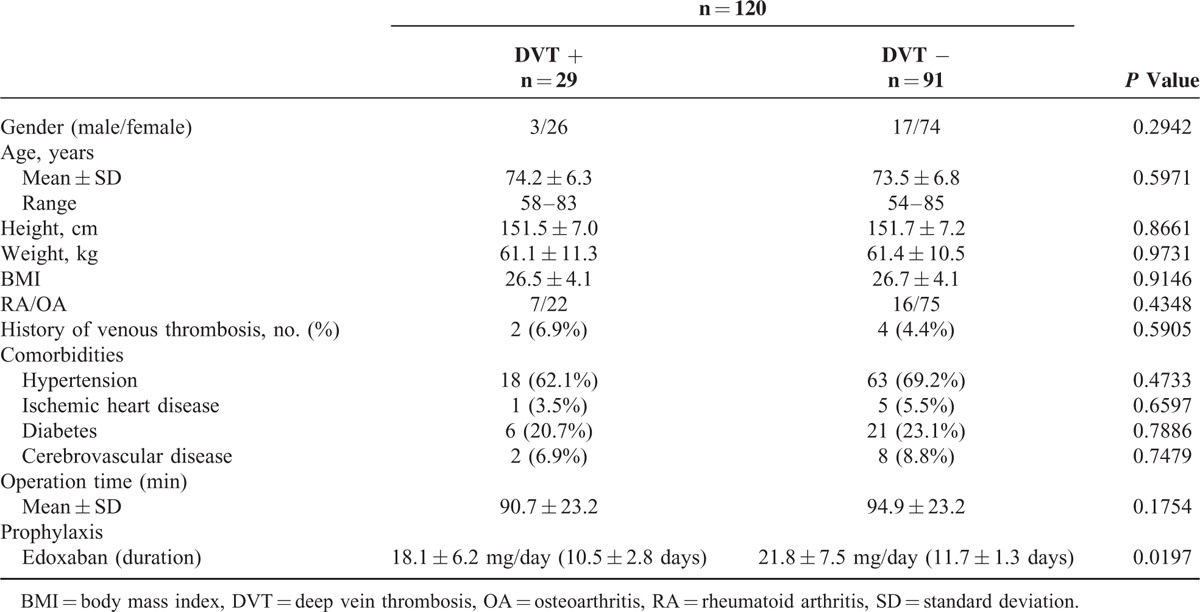
Baseline Characteristics of the Patients With or Without DVT

**TABLE 5:**
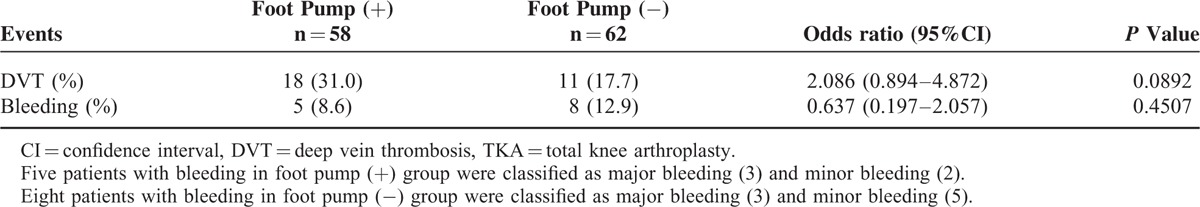
Incidences of any DVT and Bleeding in Patients Receiving TKA

### Incidence of Postoperative Bleeding

Safety analysis showed that the incidences of major bleeding up to POD28 in patients with or without the foot pump were 5.1% (n = 3) and 4.8% (n = 3), respectively. No fatal bleeding was observed (Table 6). Among the 6 patients with major bleeding, edoxaban administration was ceased at POD4 in 1 patient. It was continued in the remaining 5 patients. We also compared the incidence of any bleeding between patients with or without the foot pump (Table 5). There was no significant difference in the incidence of bleeding between these 2 groups (*P* = 0.451; OR 0.64, 95%CI 0.20−2.06).

**TABLE 6:**
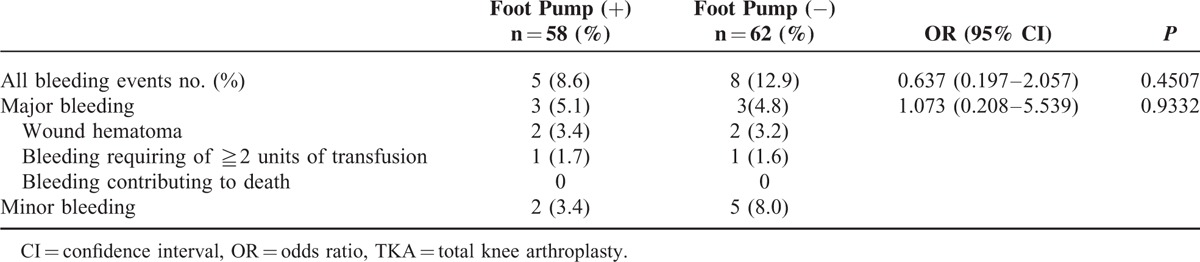
Incidences of Bleeding in Patients Receiving TKA

### Changes in Plasma D-Dimer Levels

As shown in Figure 2, preoperative D-dimer levels (mean ± SD) did not differ significantly in patients with the foot pump (1.7 ± 1.9 μg/mL) and those without the foot pump (2.4 ± 3.3 μg/mL). However, the D-dimer levels were significantly elevated on POD10 compared with those on POD0, although there was no significant difference in postoperative D-dimer levels between patients with (8.2 ± 3.8 μg/mL) or without (9.5 ± 7.2 μg/mL) the foot pump.

### Seroconversion of IgG-Class PF4/Heparin Antibody

**FIGURE 3:**
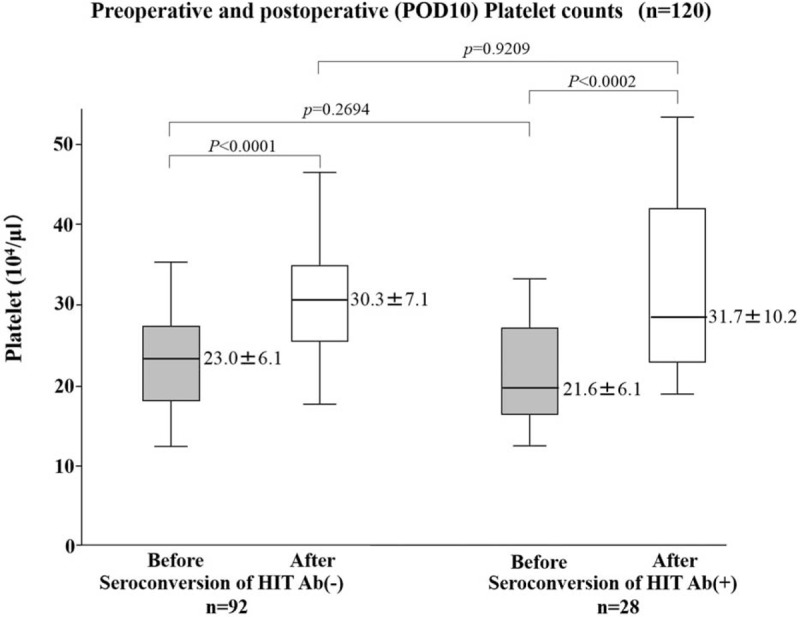
Preoperative (before) and postoperative day 10 (after) platelet counts in patients with or without the seroconvertion of HIT antibodies. *P* values were calculated by the Mann–Whitney *U* test.

The seroconversion of anti-PF4/heparin antibody was confirmed in 20.7% (12/58) of patients with the foot pump and in 25.8% (16/62) of those without the foot pump under thromboprophylaxis with edoxaban (Table 7). There was no difference in the seroconversion rate between patients with or without the foot pump. The seroconversion of anti-PF4/heparin antibodies was not significantly associated with the occurrence of DVT or bleeding (Table 8). There was no significant difference in postoperative platelet counts between patients with or without seroconversion of anti-PF4/ heparin antibodies (Figure 3). Additionally, no patients had postoperative thrombocytopenia (<10.0 × 10^4^/mL) as well as definitive heparin-induced thrombocytopenia (data not shown).

**TABLE 7:**
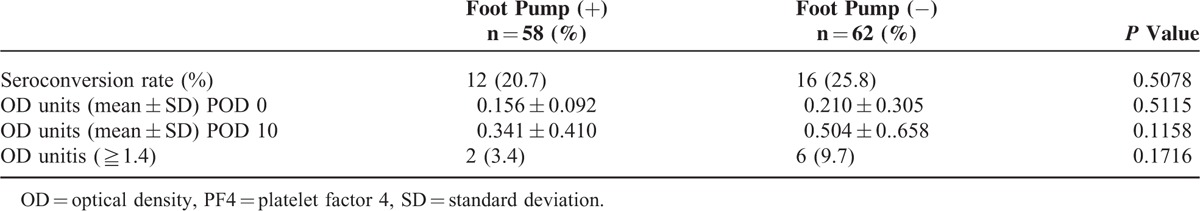
Serconvertion Rates of Anti-PF4/Heparin Antibodies Patients With or Without Foot Pump

**TABLE 8:**
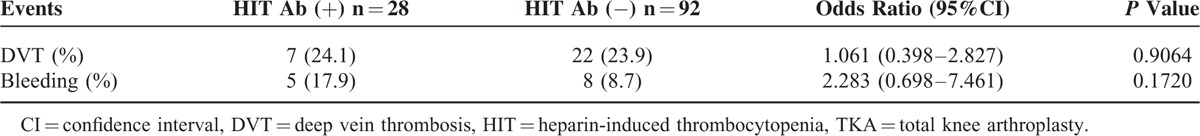
Incidences of Any DVT and Bleeding in Patients Receiving TKA

## DISCUSSION

Patients who undergo orthopedic operations such as a total joint replacement on the lower extremityare at high risk for developing thromboembolic disease.^15^ Without prophylaxis after total knee replacement, the prevalence of DVT has been reported to be as high as 50%.^16^ PE has been documented in 2% to 5% of patients not treated prophylactically, and fatal PE has been reported in 1% to 2%.^17^ With such high morbidity and mortality rates associated with thromboembolic disease, a prophylactic regimen after total knee replacement is essential.^18^ In recent years, there has been significant progress toward more effective and practical thromboprophylaxis for patients undergoing joint replacement surgery, including LMWHs and oral agents (selective factor X inhibitors) such as rivaroxaban, apixaban, and edoxaban.^10,19–21^ Also, the use of mechanical devices such as a venous calf or foot pump—either alone or in combination with chemical prophylaxis—can reduce the rate of venous thromboembolism.^22^ Mechanical devices such as the venous foot pump have been shown to be effective methods of prophylaxis.^23^

In this study, we evaluated the prophylactic effect of pneumatic foot compression under thromboprophylaxis with edoxaban in patients undergoing TKA. We found that the incidence of DVT after TKA in the combined prophylaxis group (A-V impulse pneumatic compression device and edoxaban) was 31.0% and that with edoxaban alone (without the foot pump) was 17.7%. Hence, no argument could be made for the effectiveness of mechanical devices as prophylaxis against postoperative DVT in patients given edoxaban. Our results were disappointing in that use of the foot pump could not reduce the incidence of DVT in combination with chemical prophylaxis (edoxaban). The incidences of any DVT in our study were relatively high compared with those in other clinical trials using edoxaban. However, adaptation of clinical trials data in highly selected patients to a “real-world” population could be tied to some difficulties. It is possible that TKA is postoperatively resistant to mechanical thromboprophylaxis. Another interpretation is that US is subject to considerable variation,^24^ although a recent systematic review suggested that US provides an accurate postoperative diagnosis of DVT in asymptomatic orthopedic patients. Previous studies suggested that mechanical and pharmacological methods applied for VTE prophylaxis are both effective and, when used in combination, have synergistic effects.^25^ Although there are a number of intermittent pneumatic compression systems, little evidence is available at present that differentiates these methods based on VTE prevention. It was demonstrated that calf−thigh pneumatic compression was more effective than plantar compression for reducing thigh swelling during the early postoperative stage.^26^ These differences in pneumatic compression devices may contribute to the different intervention-related outcomes.

Kakkos *et al* reported that combined intermittent pneumatic leg compression and pharmacological prophylaxis significantly reduced the incidence of DVT compared with leg compression or pharmacological prophylaxis alone. The efficacy of intermittent pneumatic compression combined with LMWH compared with LMWH alone was demonstrated in patients undergoing total hip arthroplasty.^27^ Meta-analysis concluded that leg compression augments the efficacy of pharmacological prophylaxis in preventing DVT in both TKA and THA.^28^ Whereas other studies could not demonstrate the effectiveness of mechanical prophylaxis in preventing DVT compared to the pharmacological prophylaxis.^29,30^ Although evidence from nonorthopedic patient populations suggests an advantage for combined mechanical and pharmacological prophylaxis, there are insufficient data to conclude whether combined modalities are better than either anticoagulants or mechanical compression used alone.

Another interesting finding in this study was that seroconversion of the IgG-class anti-PF4/heparin antibody occurred in a substantial number of patients under edoxaban prophylaxis. Fondaparinux, another Xa inhibitor, is occasionally associated with anti-PF-4/heparin antibodies.^32^ It was demonstrated that the frequency of forming anti-PF4/heparin antibodies was the same for patients receiving fondaparinux or enoxaparin.^31^ However, HIT rarely develops in patients on fondaparinux.^32^ The failure of these antibodies to cause HIT has been attributed to the inability of fondaparinux to react with PF4.^33^ Previously, we reported that the seroconversion rate of anti-PF4/heparin antibodies was 24.5% in patients given fondaparinux for pharmacological thromboprophylaxis.^34^ Compared with these data, the seroconversion rate of anti-PF4/heparin antibodies (23.5%) in patients given edoxaban for pharmacological thromboprophylaxis are close to those in patients given fondaparinux. The seroconversion of the IgG-class anti-PF4/heparin antibody, however, seemed not to be a risk factor for DVT or HIT in patients given edoxaban. Because of their molecular structures as Xa inhibitors, these newer anticoagulants could not interact with PF4 or be subjected to PF4 binding to platelets.^35^ Alternatively, edoxaban is a bystander, which together with other postoperative negatively charged polyanions, such as heparin sulfate, trigger an immune reaction and production of anti-PF4-heparin antibodies.^36^

## LIMITATIONS

One major limitation of the present study is that this quasi-randomized controlled trial may have several biases. It is impossible to blind patients or clinicians when using physical interventions. For sample size calculation, we used our previously published data from Japanese patients under recent thromboprophylaxis to determine a DVT rate of ∼24.3% following TKA.^37^ Based on these assumptions, we primarily calculated that we needed 126 patients with the foot pump and 126 patients without it to confirm a reduction in DVTs (relative risk 0.43) as demonstrated in a recent system review^39^ at an α error of 0.05 and a β error of 0.80. However, we could not accumulate a sufficient sample size, and there is an undeniable possibility of the study being underpowered. The credibility of our finding that the foot pump was not effective for preventing DVTs in patients who had undergone TKA should be assessed in light of current evidence.^38^ According to the Bayesian credibility assessment by Matthews *et al*,^39^ to determine the credibility of our findings in the light of existing evidence, the odds ratio had to be <0.96 before the results of our study could be considered credible. However, the calculated odds ratio in the present study was 2.1 and there was significant discrepancy. We are also concerned about the insufficient sample size.

Additional limitations include the lack of sham devices and placebo use in the study. Also, the duration of mechanical prophylaxis was relatively limited (4 days) in our study because most of the enrolled patients underwent early mobilization. Most asymptomatic DVTs detected by ultrasonography were located distally, for which the diagnostic accuracy of ultrasonography could be lower than for proximal DVT. However, ultrasonography yielded a better diagnostic performance even in patients with asymptomatic DVT when performed by trained sonographers under standardized examination procedures.

In conclusion, our randomized controlled study demonstrated that combined pharmacological (edoxaban) and mechanical prophylaxis (a foot pump) did not reduce the incidence of DVT in patients who had undergone TKA surgery compared with edoxaban alone. Further research should focus on the efficacy of more recent intermittent compression with anticoagulants, such as factor Xa inhibitors. Seroconversion of anti-PF4/heparin antibodies was detected in a substantial number of patients who underwent TKA and who were under antithrombotic prophylaxis using edoxaban.
